# The association between believing staying active is beneficial and achieving a clinically relevant functional improvement after 52 weeks: a prospective cohort study of patients with chronic low back pain in secondary care

**DOI:** 10.1186/s12891-020-3062-6

**Published:** 2020-01-20

**Authors:** Allan Riis, Emma Louise Karran, Janus Laust Thomsen, Anette Jørgensen, Søren Holst, Nanna Rolving

**Affiliations:** 1 Center for General Practice at Aalborg University, Fyrkildevej 7, 1. sal, 9220 Aalborg, Denmark; 2 School of Health Sciences, University of South Australia, GPO Box 2471, Adelaide, South Australia 5001 Australia; 3 Diagnostic Centre, Silkeborg Regional Hospital, Falkevej 1-3, 8600 Silkeborg, Denmark; 4 DEFACTUM, Central Denmark Region, Aarhus, Denmark

**Keywords:** Low Back pain, Referral and consultation, Diagnostic imaging, Patients’ beliefs, staying active, Functional improvement

## Abstract

**Background:**

According to clinical guidelines, advice to stay active despite experiencing pain is recommended to patients with non-specific low back pain (LBP). However, not all patients receive guideline-concordant information and advice, and some patients still believe that activity avoidance will help them recover. The purpose was to study whether guideline-concordant beliefs among patients and other explanatory variables were associated with recovery. The main aim was to investigate whether believing staying active despite having pain is associated with a better functional outcome.

**Methods:**

This was a prospective cohort study involving adults with non-specific LBP referred from general practices to the Spine Centre at Silkeborg Regional Hospital, Denmark. Patients reported on their beliefs about the importance of finding the cause, the importance of diagnostic imaging, perceiving to have received advice to stay active, pain duration, pain intensity, and STarT Back Tool. Agreeing to: ‘An increase in pain is an indication that I should stop what I’m doing until the pain decreases’ adjusted for age, gender, and education level was the primary explanatory analysis. A 30% improvement in the Roland Morris Disability Questionnaire (RMDQ) score after 52 weeks was the outcome.

**Results:**

816 patients were included and 596 (73.0%) agreed that pain is a warning signal to stop being active. Among patients not considering pain as a warning signal, 80 (43.2%) had a favourable functional improvement of ≥30% on the RMDQ compared to 201 (41.2%) among patients considering pain a warning signal. No difference was found between the two groups (adjusted *P* = 0.542 and unadjusted *P* = 0.629). However, STarT Back Tool high-risk patients had a less favourable functional outcome (adjusted *P* = 0.003 and unadjusted *P* = 0.002). Chronic pain was associated with less favourable functional outcome (adjusted *P* < 0.001 and unadjusted P < 0.001), whereas beliefs about finding the cause, diagnostic imaging, perceiving to have received advice to stay active, or pain intensity were not significantly associated with outcome.

**Conclusions:**

Holding the single belief that pain is a warning signal to stop being active was not associated with functional outcome. However, patients characterised by having multiple psychological barriers (high-risk according to the STarT Back Tool) had a less favourable functional outcome.

**Trial registration:**

Registered at ClinicalTrials.gov (registration number: NCT03058315), 20 February 2017.

## Background

The one-month prevalence of low back pain (LBP) is estimated to be 23.2%, and many individuals with LBP consult their general practitioner for treatment [1, 2]. In accordance with international guidelines for the management of LBP, healthcare professionals are expected to routinely provide advice to stay active [3–6]. However, this advice is inconsistently delivered [7]. Some primary care healthcare professionals believe that avoidance of activities and work will help the patient to recover [8], and as a consequence, their patients are also likely to believe that inactivity will facilitate recovery [9]. In addition, negative expectations for recovery, fear-avoidance beliefs, and catastrophic thoughts about their back pain may drive patients to request scans, diagnostics, and other secondary care specialist services [10]—often in conflict with guideline-concordant care.

The information and recommendations healthcare professionals provide about LBP to their patients may differ from what their patients perceive they have been told [11]. Moreover, what patients *perceive* to have been recommended may differ from what they actually *believe* will help them recover [11]. Thus, changing patients’ beliefs is considered a crucial factor for changing actual behaviour and has the potential to impact functional outcomes [12]. Effective delivery of information about the importance of remaining active and the patients’ role in self-management has been shown to reduce the utility of primary healthcare and reduce referrals to more expensive treatments in secondary care [13, 14]. Patients with multiple psychological barriers for recovery who are classified as ‘high risk’ according to The STarT Back screening tool are expected to have a poorer outcome than patients not in the high-risk group [14, 15]. More complex care may be indicated for this high-risk group—including specialist consultation—such that their identification was considered relevant to this study. Addressing psychological patients’ beliefs about staying active is likely to assist in individually tailoring the information and advice provided during an LBP consultation—leading to cost savings and improved treatment outcomes [4–7]. Furthermore, clinical guidelines include advice to stay active, do not recommend performing x-rays and MR scans to rule in, and recommend informing patients that finding the cause is often not relevant for recovery [6]. The importance of delivering guideline-concordant information and the importance of patients’ beliefs are, however, poorly understood [16]. Consequently, the overall purpose was to study whether guideline-concordant beliefs among patients and guideline-concordant information and advice provided by health care professionals are associated with better patient outcomes. To the best of our knowledge, this has not been studied prospectively in a population of patients with LBP being referred from primary to secondary care.

### Aims

The main aim of this study was to investigate whether believing staying active despite having pain is associated with better functional outcomes among patients referred from general practice to an outpatient spine clinic in a secondary care setting in Denmark. Additional aims were to study the association between having received advice from a health professional to stay active, patients’ pain intensity, pain duration, and the STarT Back Tool and functional recovery. We hypothesised that patients who believe that staying active will help them recover would have higher odds of a 30%-improvement in the Roland Morris Disability Questionnaire (RMDQ) score after 52 weeks compared to patients who do not believe that staying active will help them recover.

## Methods

This is a prospective cohort study with a 52 weeks follow-up. Reporting follows the STROBE guidelines for observational studies in epidemiology [17]. Data were collected from a consecutive series of adults with LBP as their predominant musculoskeletal complaint, referred from general practice to the regional Spine Centre at Silkeborg Regional Hospital. In Denmark, general practice is the first-line provider of healthcare and the gatekeeper to in- and outpatient hospital treatment [18]. All patients referred to the Spine Centre receive a digital letter with a link to an online questionnaire to be completed approximately one week before their appointment at the Spine Centre. To be eligible for this study, patients needed to be referred to the Silkeborg Spine Centre and have completed the electronic questionnaire.

### Inclusion criteria

1. ≥ 18 years of age at the time of completion of the baseline questionnaire.

2. LBP is the primary cause of the referral to the Spine Centre.

### Exclusion criteria

1. Known spinal fractures, infection, or inflammatory disease.
2. The LBP is suspected to be caused by malignancy.
3. Unwilling to participate.

### Data collection

All patients referred to the Spine Centre are routinely provided with a standard electronic questionnaire one week prior to their appointment. Patients reporting LBP as their primary complaint were provided with further information about voluntary involvement in this study and were asked whether they wished to participate. All data were self-reported in questionnaires. Patients could, at any time and without any consequence for their treatment, discontinue their participation. Consenting patients were requested to reply to extra questions in addition to the standard questionnaire and to complete another questionnaire after 52 weeks. Patients not responding to the 52-week questionnaire were sent reminder emails after two and three weeks and delivered postal reminders after four weeks. Patients who still did not respond were contacted by telephone.

The majority of questions included in the baseline questionnaire have been routinely administered since January 2016, with some adjustments following clinician-patient feedback to NR. The new questions included in the baseline questionnaire and the questions in the follow-up questionnaire were tested for face validity on 10 patients referred to the Spine Centre prior to this study. This testing process involved the patients completing the questionnaire with NR present and offering verbal feedback. This feedback led to minor question modifications. The RMDQ remained unchanged.

### Baseline questionnaire

Patients were asked to rate their agreement with the following eight questions (explanatory variables). The primary explanatory was item 9 in the short form of the Örebro musculoskeletal pain screening questionnaire [19]. The validated Danish version of the item was applied [20, 21]:

1. ‘An increase in pain is an indication that I should stop what I’m doing until the pain decreases.’ (Mark one of 11 boxes displayed on a horizontal line (0–10 points), a higher score indicating higher agreement).

The scales were labelled 0 (do not agree) at the left side of the line and 10 (totally agree) at the right side. Scores of 0–5 were regarded as ‘disagreement’ and were coded 0; scores of 6–10 were regarded as ‘agreement’ and were coded 1.

### Secondary explanatory variables were:

2) ‘I think that finding the cause of pain is important for my recovery’ (0–10). Scores of 0–5 were regarded as ‘disagreement’, and scores of 6–10 were regarded as ‘agreement’.
3) ‘I think x-rays and MR scans are an important part of my recovery’ (0–10). Scores of 0–5 were regarded as ‘disagreement’, and scores of 6–10 were regarded as ‘agreement’.
4) ‘Have you been advised by your general practitioner to stay active despite your back pain?’ (y/n).
5) Have you been advised by a physiotherapist or chiropractor to stay active despite your back pain?’ (y/n).
6) Pain duration (≥ 12 weeks, y/n).
7) Numerical pain rating (0–10) [22].
8) The STarT Back Tool (High risk, y/n) [23]. The STarT Back Tool is a patient-reported questionnaire, which allocates respondents with LBP to low-risk, medium-risk, or high-risk categories based on nine items [23]. Patients in the high-risk group are characterised by their holding a combination multiple psychological barriers (beliefs) to recovery (items 5–9), for example, being fear-avoidant, having worrying thoughts about recovery, having a low mood, and/or catastrophising [23].

Other included baseline variables: Health-related quality of life (EQ-5D-3 L) [24], age, gender, employment status, sick leave, educational level, smoking status, questions related to co-morbidity (previous surgery and present disease), and the baseline RMDQ [25].

### Outcome

The outcome was the proportion of patients indicating a clinically relevant improvement on the RMDQ score after 52 weeks, adjusted for age, gender, and educational level. A threshold of 30% improvement between baseline and 52 weeks was considered a clinically relevant improvement [26]. This was based on a study from the UK, in which a 30% improvement in RMDQ between baseline and follow-up defined the clinically relevant improvement in function [27].

### Statistical analysis

**Fig. 1:**
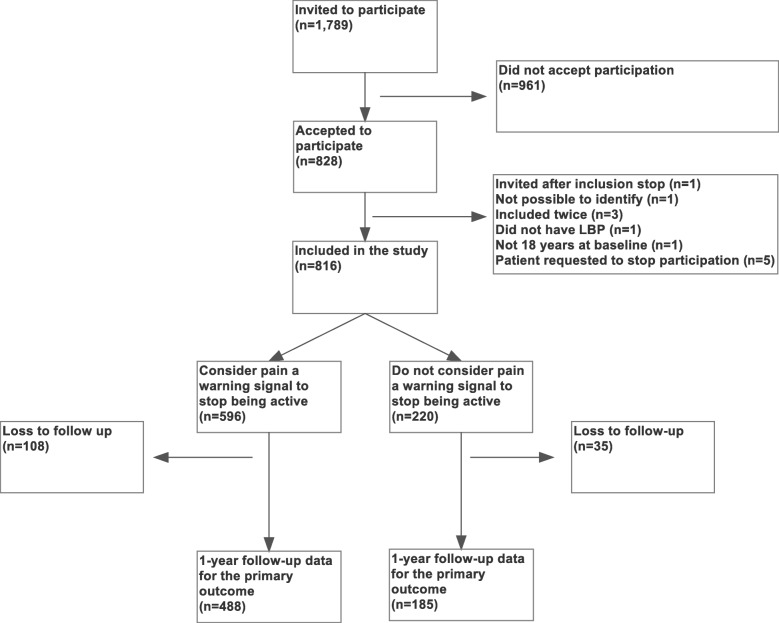
Flowchart. A consecutive cohort of 1789 patients with low back pain (LBP) was invited to participate after they filled out a routinely administered questionnaire online before their appointment at the Diagnostic Centre at Silkeborg Region Hospital, Denmark in 2017

Reporting of baseline characteristics includes numbers (%) for categorical variables and means (SD) or medians (IQRs) for continuous variables depending on the distribution of the data. Responses (0–5 or 6–10) to the question ‘An increase in pain is an indication that I should stop what I’m doing until the pain decreases’ was considered the primary explanatory variable. Responses to ‘I think that finding the cause of pain is important for my recovery’, ‘I think x-rays and MR scans are an important part of my recovery’, ‘Have you been advised by your general practitioner to stay active despite your back pain?’, Have you been advised by a physiotherapist or chiropractor to stay active despite your back pain?’, pain duration (≥ 12 weeks), numerical pain rating (0–10), and the STarT Back Tool (high-risk group) were all considered secondary explanatory variables. The outcome (RMDQ) was adjusted for possible confounders (age, gender, and educational level) in a logistic regression analysis and analysed unadjusted in logistic regression models estimating the odds of clinically relevant improvement (30%) in the RMDQ score (Fig. 1). A statistical analysis plan was published 18 August 2017 (before administration of follow-up questionnaires) [28]. All explanatory variables are reported as adjusted (age, gender, and educational level) and unadjusted odds ratios, supported by numbers of patients with clinically relevant improvements [28]. In addition, a post hoc analysis combining the adjustment for age, gender, and level of education with baseline RMDQ, pain duration, pain intensity, smoking, employment, and comorbidities was conducted. The results were considered significant when *P* < 0.01. Analyses were performed in Stata (IC version 15.1) (College Station, Texas, USA). This study is registered at ClinicalTrials.gov (registration number: NCT03058315).

### Missing values

Imputation of missing data for explanatory variables was a priori planned using Stata’s multiple imputation routine with 20 imputations if the total of missing data was below 10% [28]. If there were more than 10% missing data, we planned to exclude non-complete observations instead of imputing the outcome variable [28].

### Power calculation

In a study from New Zealand, 80% of patients with LBP agreed with ‘If you have back pain you should try to stay active‘, and respondents held more positive views about activity if they had consulted a health professional [29]. In our sample, all patients were referred from primary care and had consulted at least one health professional. Consequently, 33% was considered a realistic estimate of the proportion of patients agreeing with: ‘An increase in pain is an indication that I should stop what I’m doing until the pain decreases’ (reply 6–10). Those predicted to disagree (reply 0–5) with ‘An increase in pain is an indication that I should stop what I’m doing until the pain decreases’ comprised 67%. Among patients replying (6–10), 50% were expected to have a clinically relevant improvement in their RMDQ score [27]. Among patients replying [0–5), 70% were expected to receive a clinically relevant improvement in their RMDQ score. With full follow-up, group sizes (33/67), alpha 0.01, and a power of 0.9; 423 patients were needed in the analysis. To account for different group sizes and loss to follow-up, 800 patients needed to be recruited.

### Handling of data

The author responsible for the analysis (AR) was blinded to the RMDQ score after 52 weeks while cleaning the dataset (baseline data). A new dataset with only baseline RMDQ and RMDQ after 52 weeks was delivered by (NR) to (AR) after follow-up was completed. This was done to ensure blinding during cleaning and coding.

## Results

Between 1 April 2017 and 22 December 2017, 1789 patients with LBP were invited to participate in this study, and 828 agreed. Of these, 816 fulfilled the inclusion criteria and were included (Fig. 1). Follow-up lasted until March 2019, with an average follow-up time of 52 weeks.

Mean age was 52.7 years (SD 13.7), and 454 (55.6%) were women. Of the participants, 739 (90.6%) had chronic LBP; the mean NPR was 5.2 (SD 2.4), and the mean RMDQ score was 14.0 (SD 4.9). Of these, 596 (73.0%) agreed that pain is a warning signal to stop being active, 717 (88.0%) considered MR scans and x-rays to be important for their recovery, and 301 (37.0%) were ‘high-risk’ patients according to the STarT Back Tool (Table 1).

**Table 1:** Baseline characteristics

	Total cohort *N* = 816	Pain is not a warning signal *N* = 220	Pain is a warning signal *N* = 596	Differences
Age, years (sd)	52.7 (13.7)	54.2 (13.8)	52.2 (13.7)	*P* = 0.068^a^
Female (%)	454 (55.6)	129 (58.6)	325 (54.5)	*P* = 0.303^b^
College-level education (%)^d^	288 (35.3)	81 (36.8)	207 (34.7)	*P* = 0.621^b^
Employed (%)^1^	728 (89.4)	205 (93.2)	523 (88.1)	*P* = 0.039^b^
Sick leave (%)	128 (15.7)	16 (7.3)	112 (18.8)	P < 0.001^b^
Current smoker (%)	138 (16.9)	31 (14.1)	107 (18.0)	*P* = 0.208^b^
History of low back surgery (%)	98 (12.0)	21 (9.6)	77 (12.9)	*P* = 0.225^b^
Co-morbidity, self-reported (%)	145 (17.8)	22 (10.0)	123 (20.6)	P < 0.001^b^
Health-related quality of life (0–1), [iqr]	0.66 [0.44; 0.78]	0.72 [0.65; 0.78]	0.66 [0.39; 0.72]	P < 0.001^c^
Roland Morris Disability Questionnaire, 0–23 points, high score = high disability, (sd)	14.0 (4.9)	11.9 (4.9)	14.7 (4.7)	P < 0.001^a^
Chronic pain (> 12 weeks = yes), (%)	739 (90.6)	196 (89.1)	543 (91.1)	*P* = 0.418^b^
Numerical Pain Rating (0–10), (sd)	5.2 (2.4)	4.6 (2.2)	5.4 (2.5)	P < 0.001^a^
STarT Back Tool, High risk, (%)^1^	301 (37.0)	42 (19.2)	259 (43.5)	P < 0.001^b^
‘I think that finding the cause of pain is important for my recovery’ (%)^2^	790 (96.9)	210 (95.9)	580 (97.3)	*P* = 0.358^b^
‘I think x-rays and MR scans are important part for my recovery’ (%)^2^	717 (88.0)	182 (83.1)	535 (89.8)	*P* = 0.015^b^
‘Have you been advised by your general practitioner to stay active despite your back pain?’, yes (%)^3^	475 (68.3)	132 (69.8)	343 (67.6)	*P* = 0.647^b^
‘Have you been advised from a physiotherapist or chiropractor to stay active despite your back pain?’, yes (%)^4^	611 (79.5)	161 (79.7)	450 (79.4)	*P* = 1.000^b^

*NOTE:* Self-reported data from patient questionnaires. ^a^Tested by the two-sample t-test. ^b^Tested by Fisher’s Exact Test. ^c^Tested by The Mann–Whitney U-test. ^d^College level education equals bachelor level. ^1^Two missing values. ^2^One missing value. ^3^122 missing values. ^4^47 missing values

Follow-up data were available for 673 (82.5%) of the patients (Fig. 1). The average improvement of all patients was 3.51 (sd 5.5) points on the RMDQ score. Among patients not considering pain a warning signal, 80 (43.2%) had a favourable functional improvement of ≥30% on the RMDQ compared to 201 (41.2%) among patients considering pain a warning signal to stop being active (Table 2). Adjusted for age, gender, and educational level, no significant difference was found between the two groups (*P* = 0.542). Without confounder adjustment, the *p*-value was 0.629.

**Table 2:** Association between explanatory variables and a favourable functional improvement after 1 year

Yes versus no	Numbers (%)	Unadjusted Odds ratio (99% CI)	P-value	Adjusted Odds ratio (99% CI)	P-value
‘Pain is a warning signal to stop physical activity’ (6–10, yes)	488 (72.5)	0.92 (0.65–1.29)	0.629	0.90 (0.57–1.42)	0.542
‘I think that finding the cause of pain is important for my recovery’ (6–10, yes)	652 (97.0)	0.71 (0.22–2.30)	0.453	0.75 (0.23–2.46)	0.538
‘I think x-rays and MR scans are an important part of my recovery’ (6–10, yes)	588 (87.5)	0.95 (0.52–1.75)	0.836	0.95 (0.51–1.76)	0.836
‘I have been advised by a general practitioner to stay active despite your back pain?’ (yes)	391 (67.3)	0.80 (0.51–1.27)	0.219	0.80 (0.50–1.28)	0.218
‘I have been advised by a physiotherapist or chiropractor to stay active despite your back pain?’ (yes)	510 (79.7)	0.99 (0.59–1.65)	0.952	0.98 (0.58–1.66)	0.933
Chronic pain (duration > 12 weeks, yes)	610 (90.6)	0.25 (0.12–0.53)	< 0.001^*^	0.25 (0.12–0.54)	< 0.001^*^
High pain (Numerical pain rating, 6–10)	320 (47.6)	0.72 (0.48–1.07)	0.033^*^	0.75 (0.50–1.13)	0.069
High risk STarT Back Tool group (yes)	237 (35.3)	0.59 (0.39–0.91)	0.002^*^	0.60 (0.39–0.94)	0.003^*^

*NOTE:* Odds for achieving a favourable outcome. Comparisons between explanatory variables and a clinically relevant improvement (≥ 30%) in the Roland Morris Disability Questionnaire (RMDQ) score after 1 year. Adjusted for age, gender, and educational level (College level). *Indicates statistically significant differences. Follow-up data was available for 673 patients (82.5%)

Among patients not considering x-rays and scans important for their recovery, 36 (42.9%) had a favourable functional improvement of ≥30% on the RMDQ compared to 245 (41.7%) among patients considering x-rays and scans important for their recovery. Adjusted for age, gender, and educational level, no significant difference was found between the two groups (*P* = 0.836).

Among patients in the STarT Back Tool medium or low-risk group, 201 (46.2%) had a favourable functional improvement compared to 80 (33.8%) among patients in the STarT Back Tool high-risk group. Adjusted for age, gender, and educational level, the difference was statistically significant (*P* = 0.003).

Pain duration was statistically significantly associated with a clinically relevant improvement in the RMDQ (*P* < 0.001). Numerical pain rating (*P* = 0.069) and advice to stay active from a GP (*P* = 0.218) or a physiotherapist or chiropractor (*P* = 0.933) were not found to be associated with an improvement in RMDQ.

## Discussion

In this observational study, patients considering pain a warning signal to stop being active did not have higher odds of achieving a less favourable functional outcome. We did find, however, that the majority of patients referred from general practice to secondary care hold erroneous beliefs that inactivity and MR scans will help their recovery (Table 1). Holding multiple erroneous beliefs, (presence of fear-avoidance, catastrophic thoughts, and depressive thoughts) and thereby being in the STarT Back Tool high-risk group [14] was associated with not achieving a clinically relevant improvement in function at 52 weeks (Table 2). This could indicate that the change of just one belief is not sufficient to improve the functional outcome of patients and a more complex psycho-behavioural approach is needed. The majority of patients had pain for three months or more. The minority of patients with shorter pain durations reported better outcomes, which is in line with previous findings [30].

### Strengths and weaknesses of the study

We applied a one-item global rating (0–10) question to measure the patients’ views on the importance of staying active (part of the Örebro questionnaire [19]). This Danish version of the Örebro has been validated as a complete questionnaire [21]; it showed acceptable measurement properties in terms of test-retest reliability and absolute reliability [21]. The applied single item was pilot tested before the trial for face validity by NR in 10 patients, who understood the question and were able to reply. Applying a cut-off between 5 and 6 was considered a practical solution and is supported by findings from Norway, where patients having a high psychosocial sub-score according to SBT expressed a median fear of working as 10 (IQR, 6–10) [31]. Visual inspection showed that the distribution was not normally distributed and did not reveal the distribution of three or more subgroups. Applying other cut-off points did not considerably change the estimates. Even though dichotomising, in general, can lead to loss of information and thereby loss of power [32], dichotomising of the primary explanatory was found optimal in our analysis. Consequently, we believe that patients’ baseline beliefs about staying active are captured in a reasonable manner with the choice of question. The follow-up percentage of above 80% is a strength of this study as it decreases the risk of retention bias. Intermediate outcome measurements between baseline and 52 weeks were not collected. This is a limitation, since intermediate measurement may have identified potential confounding from recurrence and new episodes. Of the 1789 patients invited, 828 (46%) accepted to participate. We did not collect consent to report on baseline characteristics among non-participants; this may limit the generalisability of the findings and is a weakness of the study. Due to the observational design, it is not possible to make any conclusions on causation, but rather to point towards an association. This appears to be supported by observing other baseline characteristics (Table 1)—patients holding different beliefs regarding activity also differed on other aspects. In the statistical analysis, we described the planned collection of data for patients’ health status (e.g., functional level and pain), behaviours (e.g., sick leave and smoking), beliefs (e.g., about staying active and importance of scans), and treatment received (e.g., receiving advice to stay active) [28]. We made an a priori decision to adjust for age, gender, and level of education [28] to avoid the risk of overcorrection of confounders by adjusting for intermediate variables [33]. However, adjusting for age, gender, and level of education together with the variables baseline RMDQ, pain duration, pain intensity, smoking, employment, and comorbidities did not significantly change the size or direction of estimates.

### Comparison with other studies

In this prospective study, we found that 73% of patients considered pain a warning signal to stop being active, which was much more than the expected 33% [28]. Furthermore, a majority of patients hold traditional biomechanical beliefs about pain, including the benefit of x-rays and MR scans. These findings are in line with the findings in a cross-sectional study, which found that patients with LBP held traditional biomedical perspectives of anatomical/biomechanical causes leading to pain [34]. Patients stated that they learned these beliefs from health professionals [34]. This finding is, however, not supported by the present study, in which the majority of patients report having been told by their clinicians to stay active. A vast majority (88%) consider MR scans important for their recovery. A scoping review of patients’ needs for medical services found that the preference for spinal imaging was driven by their desire for a diagnosis but also by the need for the legitimisation of their symptoms [35].

In the STarT Back Tool questions, 5–9 comprise the psychosocial score that determines the high-risk classification [14]. Only 37% of patients in this study were in the STarT Back Tool high-risk group, which was less than expected. In a study among patients managed in Danish general practice, the proportion of patients in the high-risk group was 32% [36]. Furthermore, in a previous study of patients with more severe symptoms, more than 50% of patients referred for fusion or decompression surgery in Denmark were in the high-risk group [37]. In contrast, only 23.6% of patients being referred to secondary care with LBP were in the high-risk group in a Norwegian study [31]. Even though patients in the high-risk group experienced a worse outcome than patients at medium or low risk, still many patients with medium risk or low risk in this study did not achieve a clinically relevant functional improvement. Consequently, all patients may have to be considered at risk, independent of their risk group allocation [38, 39].

### Meaning of the study: possible mechanisms and implications for clinicians or policymakers

A high percentage of these patients referred to secondary care for LBP held erroneous beliefs that may hinder their recovery. According to clinical guidelines for the management of LBP [3–6], primary healthcare providers should address these beliefs at an early stage. Moreover, most patients in this study reported being advised to stay active by their general practitioner (GP), physiotherapist, or chiropractor, but they still considered inactivity helpful and erroneously regarded MR scans to be important for their recovery. Possibly just believing staying active is helpful for recovery is not enough to change outcomes, and a more complex psycho-behavioural approach may be required to improve patient outcomes. This is supported by previous studies from UK general practice indicating that targeting patients’ beliefs moderates the effect of clinicians’ reassurance [40] and that the implicit reassurance provided by GPs is of importance [41]. Furthermore, it was demonstrated that patients needed to be explicitly reassured that they are being taken seriously by their GP, that the GPs want to help them, and their GPs are available to them [41]. Meeting these needs has been demonstrated to be achievable through relationship building [41]. Advice to stay active is a cornerstone of clinical guidelines [3–6], yet the effects of such advice are unclear. This study indicates that a single belief about physical activity and recovery cannot predict patients’ prognosis in terms of functional ability (RMDQ) among patients in general practice referred to hospital care. Instead, having a range of unhelpful beliefs, as measured with the STarT Back Tool, seemed to be more closely associated with functional capacity after 52 weeks. This study emphasises the need to address multiple psychological beliefs including patients’ fear-avoidant beliefs to obtain better treatment effects in clinical practice and suggests that simply delivering the advice to stay active might not change the functional outcome.

### Unanswered questions and future research

Shifting patients’ pain beliefs from the traditional biomedical perspective that pain is an indicator of tissue damage and towards a perspective (based on a contemporary understanding of pain) that supports staying active despite pain has previously been found to be difficult [34] and is likely to be hindered by the beliefs of the clinicians they encounter. A previous study has found moderate evidence that health care professionals with a biomedical orientation or elevated fear-avoidance beliefs themselves are more likely to advise patients to limit physical activities [29], which in turn can affect patients’ outcomes [42]. Consequently, there is a future need for interventions aimed at addressing clinicians’ beliefs about LBP and the benefits of staying active, with a focus on building clinical skills to optimise reassurance [43]. This may enable healthcare professionals to identify and effectively address factors such as fear-avoidance, self-efficacy, catastrophising, and recovery expectations [9, 10] that may importantly influence LBP recovery.

## Conclusion

Patients with LBP frequently hold erroneous beliefs that may be detrimental for their recovery. However, considering pain a warning signal was not associated with a favourable functional improvement. In addition, guideline-concordant beliefs about scans and x-rays, having been advised to stay active by a healthcare professional, and patients’ levels of pain were not associated with a favourable functional improvement. However, having chronic pain and belonging to the high-risk group, according to the STarT Back Tool, were associated with worse functional outcomes.

## Data Availability

The datasets used and/or analysed during the current study are available from the corresponding author on reasonable request.

## Abbreviations

Eq-5D-3 L: Euroqol 5 dimensions (3 levels) measurement of health-related quality of life
GP: General practitioner
IQR: Interquartile range
LBP: Low back pain
LBPRS: Low back pain rating scale
NRS: Numerical rating score
SD: Standard deviation
